# Targeting eEF1A1 With Baicalein to Block eEF1A1/Prdx4 Interaction for Treating Sepsis‐Mediated Lung Injury

**DOI:** 10.1002/exp2.70206

**Published:** 2026-08-24

**Authors:** Jing Chang, Hui‐Lin Zhang, Qi‐Meng Zhu, Si‐Wen Hui, Xin‐Rong Xu, Mahmood Brobbey Oppong, Yan‐Li Feng, Juan Zhang, Cheng‐Peng Sun, Feng Qiu

**Affiliations:** ^1^ School of Chinese Materia Medica, School of Medical Technology, Tianjin Key Laboratory of Therapeutic Substance of Traditional Chinese Medicine Tianjin University of Traditional Chinese Medicine Tianjin People's Republic of China; ^2^ Department of Pharmaceutical Chemistry School of Pharmacy, College of Health Sciences University of Ghana Accra Ghana

**Keywords:** baicalein, eEF1A1, interaction, lung injury, Prdx4, sepsis

## Abstract

Sepsis is a systemic inflammatory response syndrome triggered by an uncontrolled host response to infection, which causes acute lung injury (ALI) and acute respiratory distress syndrome (ARDS). Baicalein (Bai) is one of the major active components of *Scutellariae Radix* for treating pulmonary diseases. However, its specific underlying molecular mechanism is unknown for treating sepsis‐mediated ALI. Herein, we found that Bai inhibited the nuclear factor kappa B (NF‐κB) pathway to suppress inflammation and oxidative stress in vitro and in vivo, alleviating the course of lung injury. Affinity chromatography revealed that Bai directly bound to eukaryotic translation elongation factor 1 alpha 1 (eEF1A1) through hydrogen bond interaction with Q108 and D110, exhibiting a dissociation constant (*K*_d_) of 336 nM. Mechanistically, Bai blocked the interaction between eEF1A1 and peroxiredoxin 4 (Prdx4) to inhibit ring finger protein 14 (RNF14)‐mediated ubiquitin‐dependent degradation of Prdx4 based on APEX2 and Co‐IP experiments. Moreover, eEF1A1 knockdown could repress inflammation, whereas its overexpression exerted an opposite effect in the in vitro experiments. Notably, Bai did not display any additional protective effect in LPS‐mediated eEF1A1 knockdown cells, while its effect was weakened in LPS‐mediated eEF1A1 overexpression cells. In addition, our study revealed that the course of sepsis‐mediated lung injury was alleviated in vivo by eEF1A1 knockdown, and no extra effects were exhibited by Bai in sepsis‐mediated eEF1A1 knockdown mice. These findings collectively suggested that targeting eEF1A1 represented a promising therapeutic strategy against sepsis‐associated lung injury through dual anti‐inflammatory and antioxidant mechanisms, particularly via Bai's role as a direct blocker disrupting eEF1A1‐Prdx4 interactions.

## Graphical abstract

Targeting eEF1A1 with baicalein disrupts the eEF1A1‐Prdx4 interaction to prevent RNF14‐mediated ubiquitination and degradation of Prdx4. This stabilizes Prdx4 to suppress NF‐κB, thereby alleviating sepsis‐induced acute lung injury.

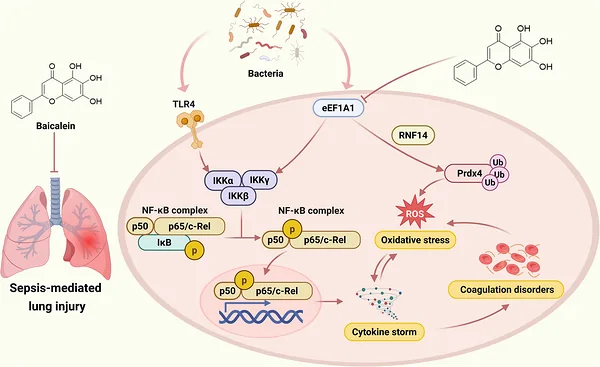

## 1 Introduction

Sepsis is a life‐threatening condition caused by bacteria, fungi, viruses, and other pathogens (such as endotoxin) that frequently leads to multiple organ dysfunction in patients in the intensive care unit (ICU) [1, 2, 3]. It often causes acute lung injury (ALI) or acute respiratory distress syndrome (ARDS) with high mortality despite current treatments [4, 5]. During sepsis, infectious insults provoke immune cell activation, eliciting inflammatory factor release and coagulation pathway engagement, thereby fostering microthrombi development and impairing tissue oxygenation [6, 7]. Under septic conditions, pathogen‐associated molecular patterns drive the nuclear factor kappa B (NF‐κB) pathway in macrophages and neutrophils, leading to the increased production of pro‐inflammatory factors such as tumor necrosis factor‐α (TNF‐α), interleukin‐1β (IL‐1β), IL‐6, and cyclooxygenase‐2 (COX‐2), amplifying the inflammatory response [8, 9, 10]. Concurrently, endothelial cells and monocytes express tissue factor (TF), initiating the extrinsic coagulation cascade and contributing to organ injury [11]. The infection also suppresses key antioxidant enzymes, while promoting reactive oxygen species (ROS) production [12, 13], thereby driving a vicious cycle of ROS and inflammation [14]. Therefore, controlling these processes is fundamental to the therapeutic intervention of sepsis‐associated lung damage.

Target fishing has revolutionized the study of effective constituents from traditional Chinese medicines by directly identifying protein interactions that explain their therapeutic effects [15, 16, 17, 18]. It has proven crucial in establishing the mechanisms of canonical drugs, ranging from artemisinin for malaria and berberine for inflammation, to more recent discoveries like wedelolactone targeting sEH for ALI, thereby providing a scientific basis for traditional knowledge [19, 20, 21, 22]. Affinity chromatography offers the distinct advantages of interrogating unmodified native compounds, enabling unbiased target discovery without prior mechanistic assumptions, and directly linking phenotypic activity to identifiable protein targets for functional validation.

Eukaryotic translation elongation factor 1 alpha 1 (eEF1A1) is a canonical protein involved in delivering aminoacyl‐tRNA to the ribosome during peptide chain elongation via GTP binding and hydrolysis [23]. Beyond its role in protein synthesis, eEF1A1 participates in diverse cellular processes, including viral infection, apoptosis, and signal transduction [24, 25, 26]. For example, plitidesin, an eEF1A blocker, exhibits a potent anti‐SARS‐CoV‐2 virus effect by inhibiting viral replication [27]. Meanwhile, emerging evidence has implicated eEF1A1 in inflammatory regulation [28, 29, 30]. For instance, silencing *eEF1A1* markedly reduces the production of multiple inflammatory factors in THP‐1 cells and BV‐2 microglial cells [28, 29]. Nevertheless, the functional mechanism by which eEF1A1 mediates the inflammatory cytokine storm in sepsis‐induced lung injury remains poorly understood.

**FIGURE 1:**
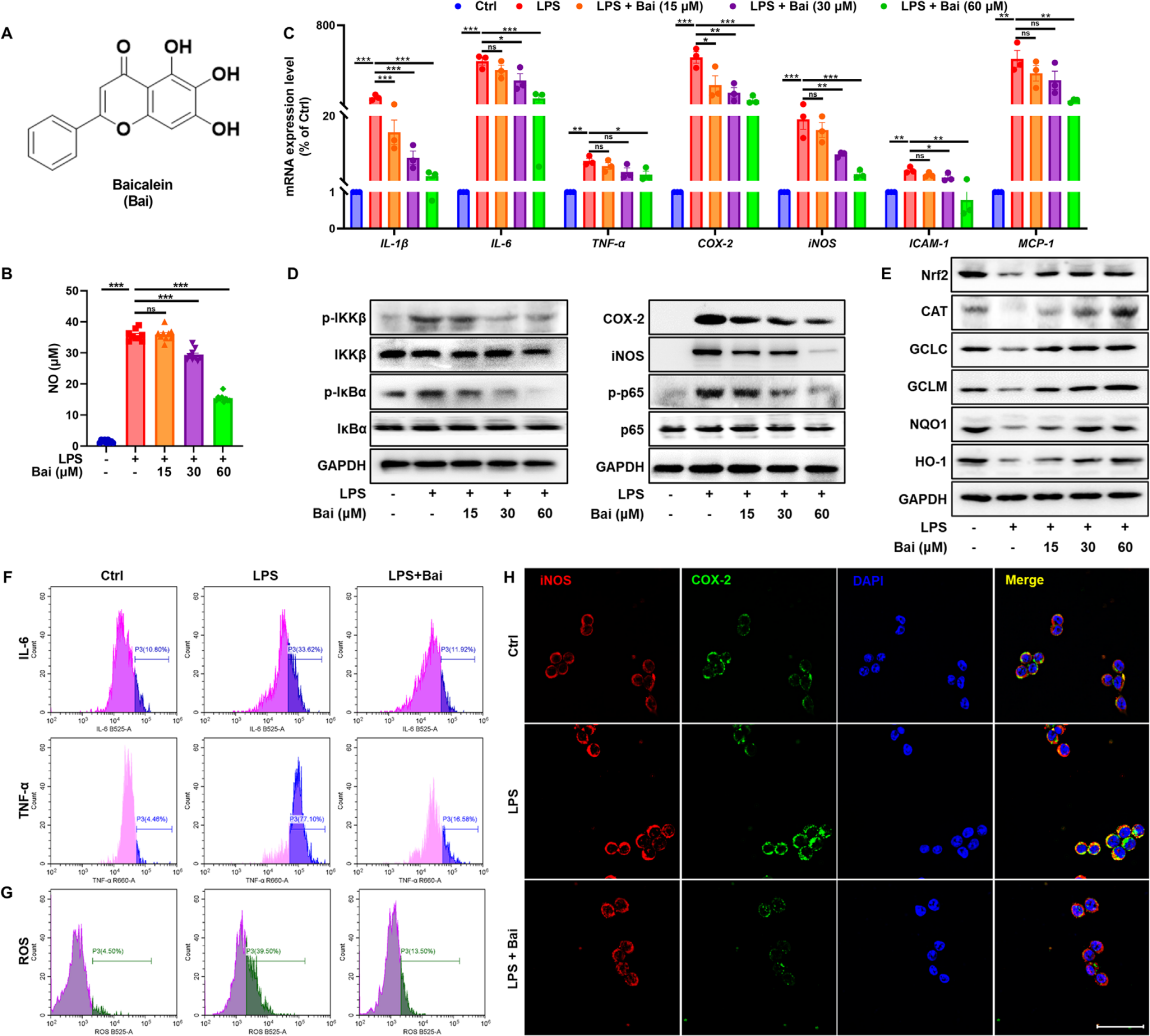
Bai exerted anti‐inflammatory and antioxidant effects via the NF‐κB pathway in vitro. (A) Chemical structure of Bai; (B) Bai significantly inhibited LPS‐stimulated NO release in RAW264.7 macrophages (*n* = 3); (C) Bai dose‐dependently suppressed the LPS‐induced mRNA expressions of key pro‐inflammatory mediators (IL‐6, IL‐1β, TNF‐α, iNOS, COX‐2, MCP‐1, and ICAM‐1, *n* = 3); (D) Bai attenuated LPS‐induced activation of the NF‐κB signaling pathway (*n* = 3); (E) Bai upregulated expressions of major antioxidant enzymes (CAT, GCLC, GCLM, NQO1, Nrf2, and HO‐1) in LPS‐challenged cells (*n* = 3); (F,G) Flow cytometry results of IL‐6, TNF‐α, and ROS (*n* = 3); (H) Representative plots of iNOS (red) and COX‐2 (green) immunofluorescence staining (*n* = 3); *p < 0.05, **p < 0.01, ***p < 0.001, ns = no significance.

Scutellariae Radix (Huang qin), a traditional Chinese medicine first documented in the “Shennong Ben Cao Jing”, has long been used for clearing heat, detoxification, hemostasis, and preventing miscarriage [31, 32, 33]. Baicalin, the principal active ingredient of Scutellariae Radix, undergoes facile conversion by intestinal flora in vivo to its aglycone baicalein (Bai, Figure 1A), subsequently mediating pronounced anti‐inflammatory actions in a wide range of disease models [34, 35]. In LPS‐induced ALI, Bai has been shown to protect against ALI by modulating NF‐κB and mitogen‐activated protein kinase (MAPK) pathways and mitigating mitochondrial dysfunction [36, 37]. Despite these findings, the precise molecular targets of Bai in sepsis‐mediated ALI remain to be clarified, highlighting the need to explore its direct binding partners and regulatory mechanisms. In this study, we systematically investigated the therapeutic potential of Bai in sepsis‐associated lung injury using both in vitro and in vivo models. We found that Bai directly bound to eEF1A1 through hydrogen bond interactions with Gln108 (Q108) and Asp110 (D110) residues, thereby inhibiting eEF1A1‐mediated ubiquitylation and subsequent degradation of peroxiredoxin‐4 (Prdx4). Collectively, this study provided direct new evidence that Bai alleviated sepsis‐induced ALI by targeting the eEF1A1–Prdx4 axis, establishing eEF1A1 as a promising therapeutic target and positioning Bai as a potential lead compound for drug development in critical care medicine.

## 2 Materials and Methods

### 2.1 Materials

The primary antibodies iNOS (22226‐1‐AP), COX‐2 (66351‐1‐Ig), p62 (A19700), CD68 (PTM5130), NQO‐1 (A1518), TNF‐α (17590‐1‐AP), IL‐6 (21865‐1‐AP), HO‐1 (A11102), GCLC (F2392), GCLM (F2395), CAT(F0722), p65 (10745), p‐p65 (AP1294), p‐IκBα (82349), IκBα (A16929), TF (AF7723), FIB (20645), p‐IKKβ (2697S), IKKβ (15649), HA (51064), His (AB18184), Gr‐1 (65078), Prdx4 (10703‐1‐AP), eEF1A1 (11402‐1‐AP), RNF14 (26368), and GAPDH (60004‐1‐Ig) were purchased from Cell Signaling Technology (CST, Danvers, MA, USA), Abcam (Massachusetts, USA), Abclonal (Wuhan, China), Abmart (Shanghai, China), PTM Bio (Hangzhou, China), Selleck (Shanghai, China), and Proteintech (Wuhan, China), respectively. Bai was isolated by us, and its purity was more than 98%, as analyzed by HPLC.

### 2.2 Cell Culture, Treatment, and Transfection

RAW 264.7 murine macrophages were grown in DMEM supplemented with 10% fetal bovine serum (FBS) at 37°C under 5% CO_2_. Cells were pretreated with Bai (15, 30, or 60 µM) before LPS (500 ng mL^−1^) stimulation. After 24 h, cells were collected for analysis.

eEF1A1 knockdown was performed using siRNA transfected with Lipofectamine 2000, while overexpression used polyethylenimine (PEI) for plasmid delivery. The overexpression of Gln108Ala (Q108A), Asp110Ala (D110A), and Lys146Ala (K146A) of eEF1A1 and Prdx4 was also transfected into cells using plasmids and the PEI method.

### 2.3 Quantitative Real‐Time Polymerase Chain Reaction (qRT‐PCR)

Total RNA from lung tissues and cells was isolated using TRIzol reagent (Agbio, China). Complementary DNA (cDNA) was synthesized using reverse transcriptase, and quantitative PCR was performed with SYBR Green. GAPDH served as the internal control. The primers were listed in Table 1.

**TABLE 1:** The sequences of primers.

Primers	Forward	Reverse
*Mouse‐IL‐6*	TTCTTGGGACTGATGCTGGT	CATTTCACCGATTTCCCAGAGA
*Mouse‐IL‐1β*	GAAATGCCACCTTTTGACAGTGA	GTCCTCATCCTGGAAGGTCC
*Mouse‐TNF‐α*	ATGGCCTCCCTCTCATCAGT	TTTGCTACGACGTGGGCTAC
*Mouse‐iNOS*	GTTTGACCAGAGGCCAGA	GTGAGCTGGTAGGTTCCTGT
*Mouse‐COX‐2*	AGGTGATTGGTGGAGAGGTG	CCTGCTTGAGTATGTCGCAC
*Mouse‐ICAM‐1*	GTCCGCTGTGCTTTGAGAACT	CGGAAACGAATACACGGTGAT
*Mouse‐MCP‐1*	TTACAGGTCAGGTCCCCTACT	CTCCTTATCCAGTATGGTCCTG
*Mouse‐PAR‐1*	TGAACCCCCGCTCATTCTTTC	CCAGCAGGACGCTTTCATTTTT
*Mouse‐EPCR*	GTAAGTTTCCGGCCAAAGACT	TCCCCTCAGACCTTTCATGTTT
*Mouse‐TF*	AACCCACCAACTATACCTACACT	GTCTGTGAGGTCGCACTCG
*Mouse‐TM*	CTCTCCGCACTAGCCAAGC	GGAGCGCACTGTCATCAAATG
*Mouse‐NQO1*	AGGATGGGAGGTACTCGAATC	CTGCAGCTTCTTCACCTCTCA
*Mouse‐HO‐1*	AAGCCGAGAATGCTGAGTTC	GCCGTGTAGATATGGTACAAGGA
*Mouse‐eEF1A1*	CAACATCGTCGTAATCGGACA	GTCTAAGACCCAGGCGTACTT
*Mouse‐GAPDH*	TCAAGATCATCAGCAATGCC	CGATACCAAAGTTGTCATGGA

### 2.4 Flow Cytometry

After pretreatment, cells were incubated with IL‐6 or TNF‐α primary antibodies at 4°C for 1 h, followed by fluorescent secondary antibodies. After washing with phosphate‐buffered saline (PBS), samples were analyzed using CytoFlex flow cytometry (Beckman, USA). For ROS detection, cells were incubated with DCFH‐DA at 37°C for 30 min and then analyzed.

### 2.5 Western Blot

Whole protein extracts were prepared in RIPA buffer supplemented with phosphatase inhibitors. Following SDS‐PAGE and transfer to PVDF membranes, standard immunoblotting was performed with primary antibodies and horseradish peroxidase (HRP)‐conjugated secondary antibodies. Chemiluminescence signals were acquired and quantified with enhanced chemiluminescence (ECL) reagents and ImageJ. Antibody working concentrations were as follows: iNOS (1:2000), COX‐2 (1:2000), p62 (1:3000), NQO‐1 (1:1000), HO‐1 (1:1000), GCLC (1:3000), GCLM (1:1000), CAT(1:1000), p65 (1:1000), p‐p65 (1:1000), p‐IκBα (1:1000), IκBα (1:10000), TF (1:2000), FIB (1:2000), p‐IKKβ (1:1000), IKKβ (1:1000), HA (1:4000), His (1:1000), Prdx4 (1:1000), eEF1A1 (1:2000), RNF14 (1:1000), and GAPDH (1:5000).

### 2.6 Immunohistochemical and Immunofluorescent Staining

For cellular immunofluorescence, cells cultured were pretreated, fixed, permeabilized, blocked with 5% BSA, and incubated with p65 (1:200), iNOS (1:200), or COX‐2 (1:200) primary antibodies at 4°C. Cells were incubated with fluorescence secondary antibodies and 4',6‐diamidino‐2‐phenylindole (DAPI), and analyzed by a Leica DM6B Thunder system.

Immunohistochemical and immunofluorescence staining were performed on paraffin‐embedded lung sections. After dewaxing, blocking endogenous peroxidase, and antigen repair of the sections, they were incubated overnight with CD68 (1:200) or Gr‐1 (1:100) at 4°C. Detection was achieved with 3,3′‐diaminobenzidine (DAB) or fluorescent secondary antibodies with DAPI, followed by imaging using a Leica DM6B Thunder system.

### 2.7 Lactate Dehydrogenase (LDH) Assay

Lung tissues were homogenized in PBS, and the supernatants were collected after centrifugation. After the concentrations of supernatants were detected by the BCA method, their LDH activities were analyzed by the corresponding kit.

### 2.8 Experimental Animal Models

Male ICR mice, weighing 20–25 g, were obtained from the Experimental Animal Center of Tianjin University of Traditional Chinese Medicine. Animals were housed under controlled conditions comprising a 12‐hour light/dark cycle, a temperature of 22°C–24°C, and 50%–65% relative humidity, with ad libitum access to standard rodent chow and water. All the experimental procedures were approved by the Animal Care and Use Committee of Tianjin University of Traditional Chinese Medicine (No. TCM‐LAEC2023208s1550). Sepsis‑associated ALI was induced by caecal ligation and puncture (CLP); the sham group underwent the same surgery without caecal puncture. Briefly, mice were anesthetized by intraperitoneal injection of tribromoethanol, the abdominal cavity was opened to locate the ileocecal valve, the cecum was ligated below the ileocecal valve, punctured once at the central position of the cecal wall with an 18‐gauge needle, and a small amount of fecal material was gently extruded. The cecum was then returned to the abdominal cavity, and the incision was sutured. CLP mice received intragastric administration of Bai (50, 100 or 200 mg kg^−1^) for 3 days, starting on the day of surgery. Sham mice received the vehicle (10% hydroxypropyl β‐cyclodextrin), and a positive control group received dexamethasone (7 mg kg^−1^, i.p.) post‐CLP. Lung tissues were collected 24 h after the final procedure.

### 2.9 Respiratory Function Test

The mouse pulmonary function was measured using an EMKA Animal Pulmonary Function Monitoring System after proper calibration in a blind test. Mice were acclimated in a plethysmography chamber for 5 min, followed by 10–20 min of data recording.

### 2.10 Hematoxylin and Eosin Staining (H&E)

Lung tissues were fixed in 4% formalin, dehydrated, paraffin‐embedded, sectioned (4 µm), and stained with H&E for histological evaluation.

### 2.11 Target Protein Fishing Assay

Epoxy‐activated Sepharose 4FF beads were coupled with Bai as in the previous method [38]. After coupling and blocking, the beads were incubated with cell lysates. Captured proteins were eluted and analyzed by silver staining, Western blot, and liquid chromatography–tandem mass spectrometry (LC‐MS/MS).

### 2.12 Cell Thermal Shift Analysis (CETSA) and Drug Affinity Responsive Target Stability (DARTS)

Cellular lysates were incubated with Bai (100 µM) or dimethyl sulfoxide (DMSO) for 2 h at room temperature. Samples were heated (47°C–59°C, 3 min), centrifuged, and analyzed by Western blot. For DARTS, lysates were incubated with Bai (25–100 µM) or DMSO, treated with pronase (3 µg mL^−1^, 37°C, 10 min), and analyzed by Western blot.

### 2.13 Microscale Thermophoresis (MST)

Labeled eEF1A1 protein was incubated with varying concentrations of Bai and Prdx4 in standard buffer for 15 min. The binding affinities (*K*_d_) between Bai and eEF1A1, eEF1A1 and Prdx4, and eEF1A1 and Prdx4 in the presence of Bai were determined using a Monolith instrument (Nano‐Temper Technologies, München, Germany).

### 2.14 Ascorbate Peroxidase 2 (APEX2) Proximity Labeling Experiment

The APEX2 experiment was conducted as previously described [39]. Cells were treated with biotin‐phenol and incubated at 37°C for 4 h. Then, cells were treated with H_2_O_2_ followed by PBS washing, collection, and lysis on ice for 2 h. These mixed protein lysates were incubated with streptavidin beads at 4°C for 2 h and then washed thoroughly. Finally, the protein complexes were digested with trypsin and analyzed by LC‐MS/MS.

### 2.15 Ubiquitylation Assay

HA‐eEF1A1, His‐Prdx4, and Myc‐ubiquitin plasmids were transfected into starved HEK293T cells using PEI. Prior to LPS treatment, cells were incubated with Bai (60 µM) and MG132 (2 µM). His‐tagged proteins were immunoprecipitated from cell lysates and analyzed by Western blot.

### 2.16 Immunoprecipitation (IP)

HEK293T cell lysates prepared in phenylmethylsulfonyl fluoride (PMSF)‐containing buffer were incubated with IgG, HA, or His antibodies at 4°C, followed by protein A/G magnetic beads. The immunoprecipitated proteins were then eluted and analyzed by immunoblotting.

### 2.17 Molecular Docking

The interaction of Bai with eEF1A1 afforded by AlphaFold was analyzed by Autodock4 and plotted by PyMOL 2.4 software as previously described [40].

### 2.18 Statistical Analysis

Statistical analyses were performed using Student's *t*‐test for two‐group comparisons, one‐way ANOVA for multiple groups with one variable, and two‐way ANOVA for designs with two variables in GraphPad Prism 9.5. All experiments were performed with biological replicates from independent experiments. All data were expressed as the mean ± standard error of the mean (mean ± SEM). The Shapiro–Wilk test was applied to assess normality. For comparisons between two groups, Student's *t*‐tests were used, or the Mann–Whitney *U*‐test if applicable. Statistical tests were selected based on data distribution, with statistical significance defined as *p* < 0.05.

## 3 Results

### 3.1 Bai Alleviated the Inflammatory Response and Oxidative Stress via the NF‐κB Pathway In Vitro

First, we performed the CCK‐8 assay to confirm that both 60 µM Bai (Figure S1A) and 4 µg mL^−1^ LPS (Figure S2) were non‐toxic to RAW264.7 macrophages. In LPS‐stimulated macrophages, Bai significantly reduced levels of inflammatory mediators, including nitric oxide (NO), IL‐6, IL‐1β, iNOS, TNF‐α, COX‐2, monocyte chemoattractant protein‐1 (MCP‐1), and intercellular adhesion molecule‐1 (ICAM‐1), and suppressed iNOS and COX‐2 protein expressions (Figure 1A–D,F; Figure S1B,C). A dose‐dependent regulatory effect was exerted via the NF‐κB pathway (Figure 1D; Figure S1C). Furthermore, cellular immunofluorescence experiments confirmed that Bai significantly suppressed the expression of COX‐2 and iNOS (Figure 1H). In addition, Bai significantly reduced the intracellular ROS level induced by LPS and upregulated multiple antioxidant enzymes, including Nrf2, CAT, GCLC, GCLM, NQO1, and HO‐1 (Figure 1E,G; Figure S1B,D), suggesting that it also possessed antioxidant functions. Consistent anti‐inflammatory and antioxidant effects of Bai were observed in MH‐S cells (Figure S3A–C). Collectively, Bai alleviated inflammation and oxidative stress mainly through the NF‐κB pathway in vitro.

### 3.2 Bai Alleviated Sepsis‐Induced Lung Injury In Vivo

**FIGURE 2:**
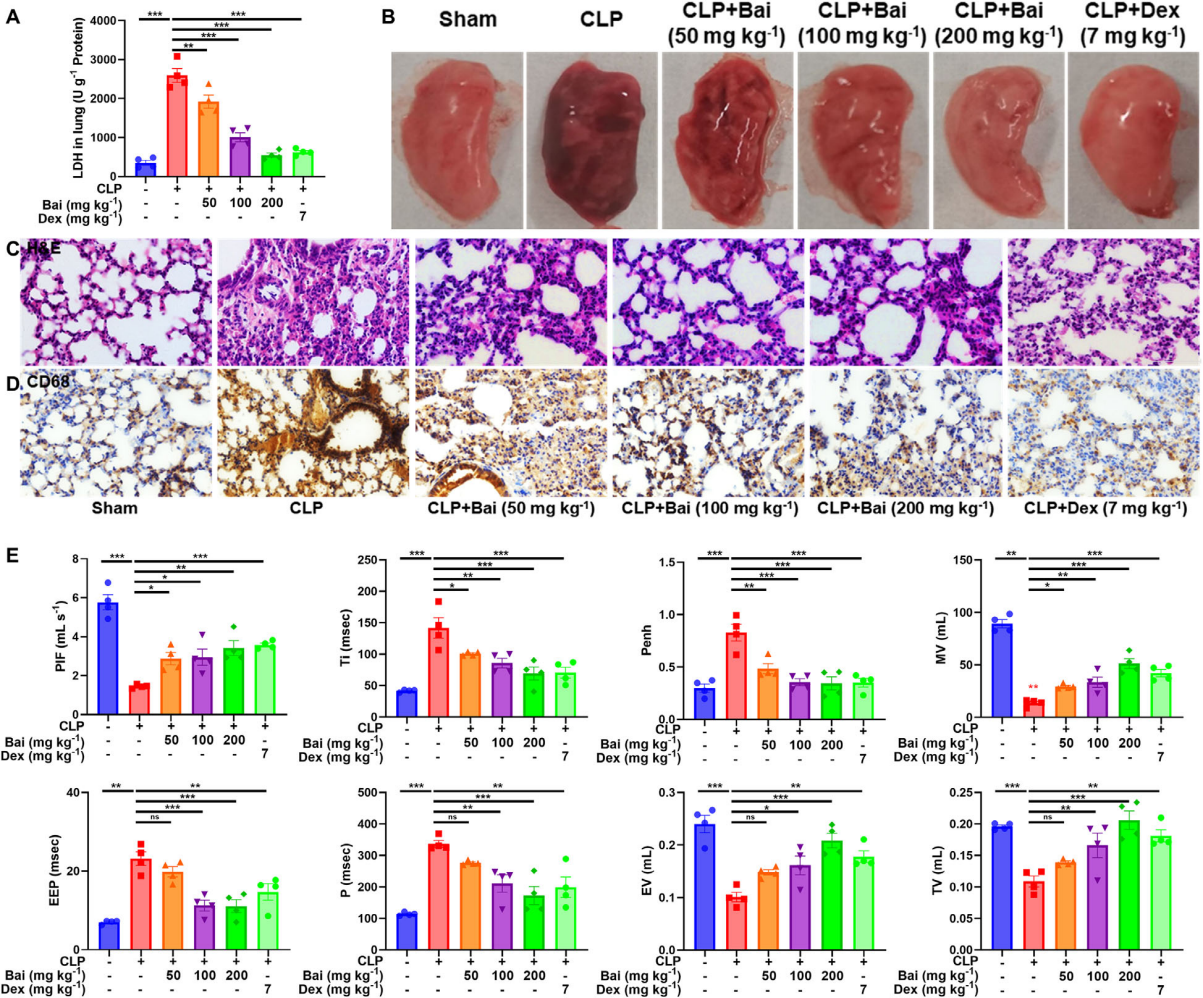
Bai alleviated sepsis‐induced lung injury in vivo. (A) Bai reduced the LDH activity in CLP‑induced sepsis (*n* = 4); (B) Representative images of lung tissues (*n* = 4); (C,D) Representative H&E and CD68 staining plots (*n* = 3); (E) Effects of Bai toward the pulmonary function (*n* = 4); *p < 0.05, **p < 0.01, ***p < 0.001, ns = no significance.

To further validate the protective role of Bai in vivo, we evaluated the effect of Bai on sepsis‐related lung injury in a CLP‐mediated mouse model. As demonstrated in Figure 2A,B, Bai treatment significantly reduced the LDH activity in lung tissue and ameliorated CLP‐induced lung tissue swelling, capillary congestion, and hemorrhage, as well as lung parenchymal structure damage. H&E staining revealed that alveolar wall thickness and alveolar collapse were increased in the CLP group, whereas these changes were improved after Bai administration (Figure 2C). Bai administration repressed the activation of macrophages (CD68, Figure 2D), and alleviated the pulmonary function of CLP‐mediated mouse through increasing peak inspiratory flow (PIF), expiratory volume (EV), minute ventilation (MV), and tidal volume (TV), and suppressing inspiratory time (Ti), airway stenosis index (Penh), end‐expiratory pause (EEP), and pause (P) (Figure 2E). Collectively, these data indicated that Bai significantly alleviated the pathological damage to lungs caused by sepsis and improved pulmonary function.

### 3.3 Bai Attenuated the Inflammation Response, Oxidative Stress, and Coagulation Dysfunction via the NF‐κB and Nrf2 Pathways in Septic Mice

**FIGURE 3:**
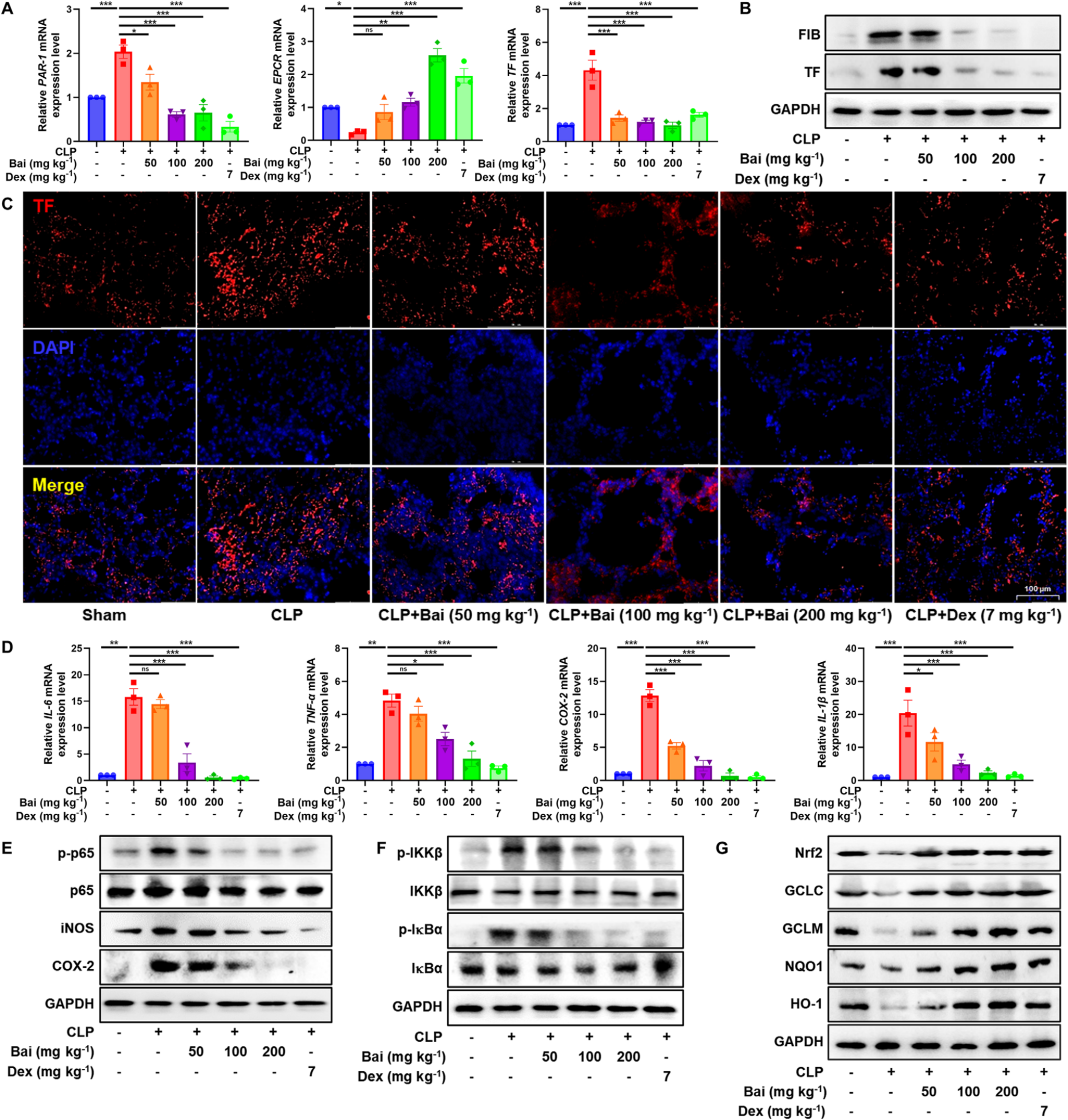
Bai attenuated inflammation, oxidative stress, and coagulation dysfunction via the NF‐κB pathway in septic mice. (A) Effects of Bai toward coagulation factors TF, PAR‐1, and EPCR in CLP‐mediated lung injury (*n* = 3); (B) Bai downregulated TF and FIB protein expression (*n* = 3); (C) Representative TF staining plots (*n* = 3); (D) Bai inhibited mRNA expressions of IL‐6, TNF‐α, COX‐2, and IL‐1β in CLP‐mediated sepsis mice (*n* = 3); (E,F) Inhibitory effects of Bai toward the NF‐κB pathway (*n* = 3); (G) Effects of Bai on oxidative stress‐related proteins Nrf2, GCLC, GCLM, NQO1, and HO‐1 in sepsis‐induced lung injury (*n* = 3); *p < 0.05, **p < 0.01, ***p < 0.001, ns = no significance.

The systemic pro‐coagulant state in sepsis drives ALI through microvascular thrombosis and endothelial damage. Compared with the CLP group, Bai significantly decreased mRNA expression of TF, protease‐activated receptor 1 (PAR‐1), and endothelial protein C receptor (EPCR), increased thrombomodulin (TM) mRNA expression, and inhibited protein expression of fibrinogen (FIB) and TF, thereby restoring coagulation dysfunction (Figure 3A–C; Figure S4A,B). Further results showed that Bai inhibited the NF‐κB signaling pathway to regulate inflammatory factors IL‐6, TNF‐α, iNOS, and COX‐2 on mRNA levels and iNOS by immunohistochemistry in CLP mice (Figure 3D–F; Figure S4D,F). As illustrated in Figure 3G and Figure S4E, Bai restored antioxidant capacity by increasing Nrf2, GCLC, GCLM, NQO1, and HO‐1. Therefore, our in vitro and in vivo results revealed the protective potential of Bai against sepsis‐induced lung injury.

### 3.4 eEF1A1 Served as the Potential Target of Bai

**FIGURE 4:**
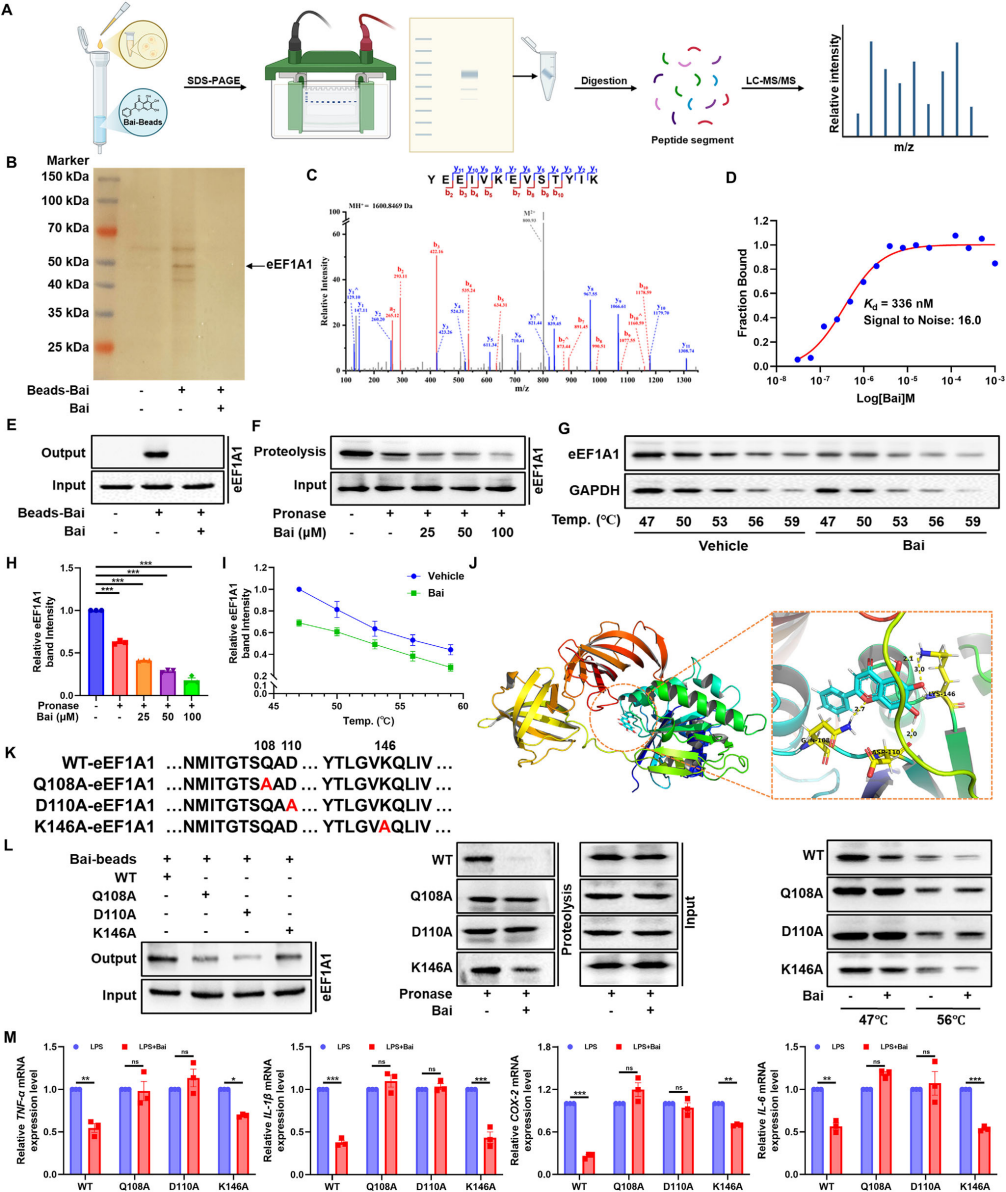
Q108 and D110 were essential for the binding of eEF1A1 with Bai. (A) Schematic diagram of affinity chromatography; (B) Sliver staining result; (C) LC‐MS/MS plot; (D) MST confirmed the high‐affinity binding between Bai and eEF1A1 (*n* = 3); (E) Pull down result (*n* = 3); (F) CETSA result; (G) DARTS result; (H,I) Quantitative data of DARTS and CETSA (*n* = 3); (J) Interactions of Bai with eEF1A1 through hydrogen bonds of Q108, D110, and K146; (K,L) Q108A and D110A mutation weakened the binding of Bai with eEF1A1 rather than K146A mutation (*n* = 3); (M) Q108A and D110A mutations abolished the anti‐inflammatory effect of Bai (*n* = 3); *p < 0.05, **p < 0.01, ***p < 0.001, ns = no significance.

To confirm potential cellular targets of Bai, it was conjugated to epoxy‐activated agarose beads (Figure 4A). A prominent ≈50 kDa band was identified as eEF1A1 via LC‐MS/MS and confirmed by immunoblotting (Figure 4B,C,E). MST assays revealed a high‐affinity binding between Bai and eEF1A1 (*K*_d_ = 336 nM), which was further validated by CETSA and DARTS experimental results (Figure 4D,F–I). As shown in Figure S5, Bai did not significantly alter eEF1A1 expression in LPS‐stimulated cells. Molecular docking illustrated that Bai bound to eEF1A1 by hydrogen bonds with Q108, D110, and K146 at a distance of 2.0–3.0 Å (Figure 4J). Mutagenesis to Q108A and D110A, but not K146A, impaired the Bai‐eEF1A1 binding and interaction stability in pull‐down, DARTS, and CETSA experiments (Figure 4K–L). Furthermore, anti‐inflammatory effects of Bai against TNF‐α, IL‐1β, COX‐2, and IL‐6 were abrogated by eEF1A1 Q108A and D110A rather than eEF1A1 K146A in LPS‐mediated RAW264.7 cells (Figure 4M), demonstrating that Q108 and D110 were critical for both the Bai–eEF1A1 interaction and its anti‐inflammatory activity.

### 3.5 eEF1A1 Knockdown and Overexpression Abolished Protective Effects of Bai In Vitro

**FIGURE 5:**
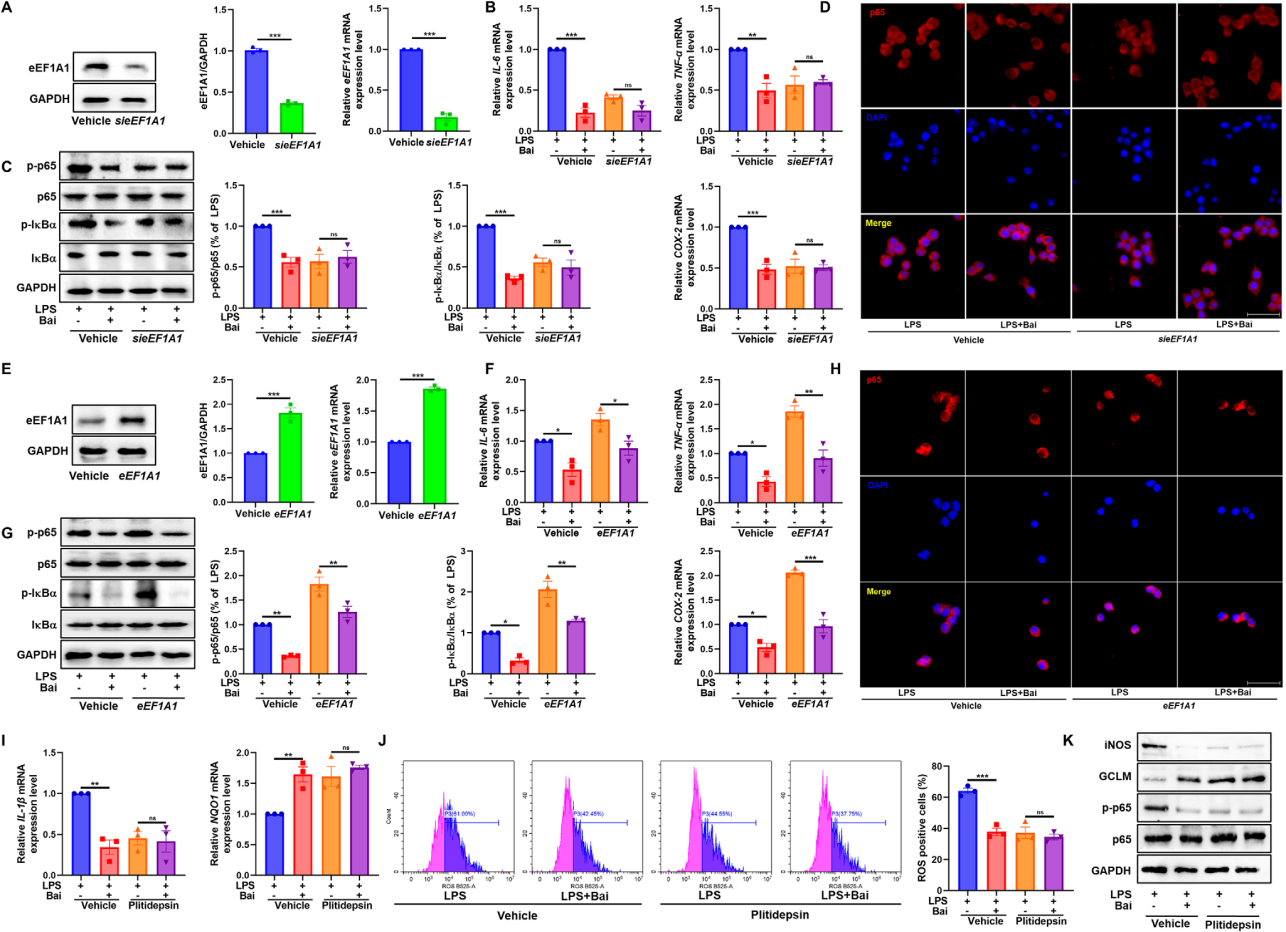
The anti‐inflammatory effect of Bai was dependent on eEF1A1. (A) Western blot and PCR validated eEF1A1 knockdown (*n* = 3); (B) eEF1A1 knockdown abolished effects of Bai on mRNA levels of IL‐6, TNF‐α, and COX‐2 (*n* = 3); (C) eEF1A1 knockdown abolished the inhibitory effect of Bai toward the NF‐κB pathway (*n* = 3); (D) Representative p65 staining plots (*n* = 3); (E) Western blot and PCR verified overexpression of eEF1A1 (n = 3); (F) eEF1A1 overexpression weakened effects of Bai toward proinflammatory genes IL‐6, TNF‐α, and COX‐2 (*n* = 3); (G) Bai‐mediated suppression effect toward the NF‐κB pathway was diminished upon eEF1A1 rescue (*n* = 3); (H) Representative p65 staining plots in LPS‐mediated normal and eEF1A1 overexpression cells (*n* = 3); (I) Plitidepsin abolished the effects of Bai on mRNA levels of IL‐1β and NQO1 in LPS‐mediated cells (*n* = 3); (J,K) Plitidepsin abolished protective effects of Bai on inflammation and oxidative stress (*n* = 3); *p < 0.05, **p < 0.01, ***p < 0.001, ns = no significance.

To validate the aforementioned findings, we generated eEF1A1‐specific knockdown and overexpression plasmids and transfected them into cells (Figure 5A,E). eEF1A1 knockdown led to a reduction in mRNA levels of IL‐6, TNF‐α, and COX‐2, decreased p‐p65 and p‐IκBα levels, and inhibited the nuclear translocation of p65 (Figure 5B–D). Notably, in LPS‐stimulated cells with eEF1A1 knockdown, the anti‐inflammatory effect of Bai was significantly attenuated. Conversely, eEF1A1 overexpression exacerbated LPS‐mediated inflammation (Figure 5F–H), while distinctly impairing anti‐inflammatory effects of Bai following rescue. Furthermore, treatment with the eEF1A1 inhibitor plitidepsin (10 nM) abrogated the effects of Bai toward inflammation and oxidative stress, such as ROS, IL‐1β, NQO1, iNOS, GCLM, and p‐p65 (Figure 5I–K; Figure S6A,B). These results demonstrated that the anti‐inflammatory effect of Bai was dependent on the target eEF1A1.

### 3.6 Bai Blocked the eEF1A1/Prdx4 Interaction to Suppress the Ubiquitylation and Degradation of Prdx4

**FIGURE 6:**
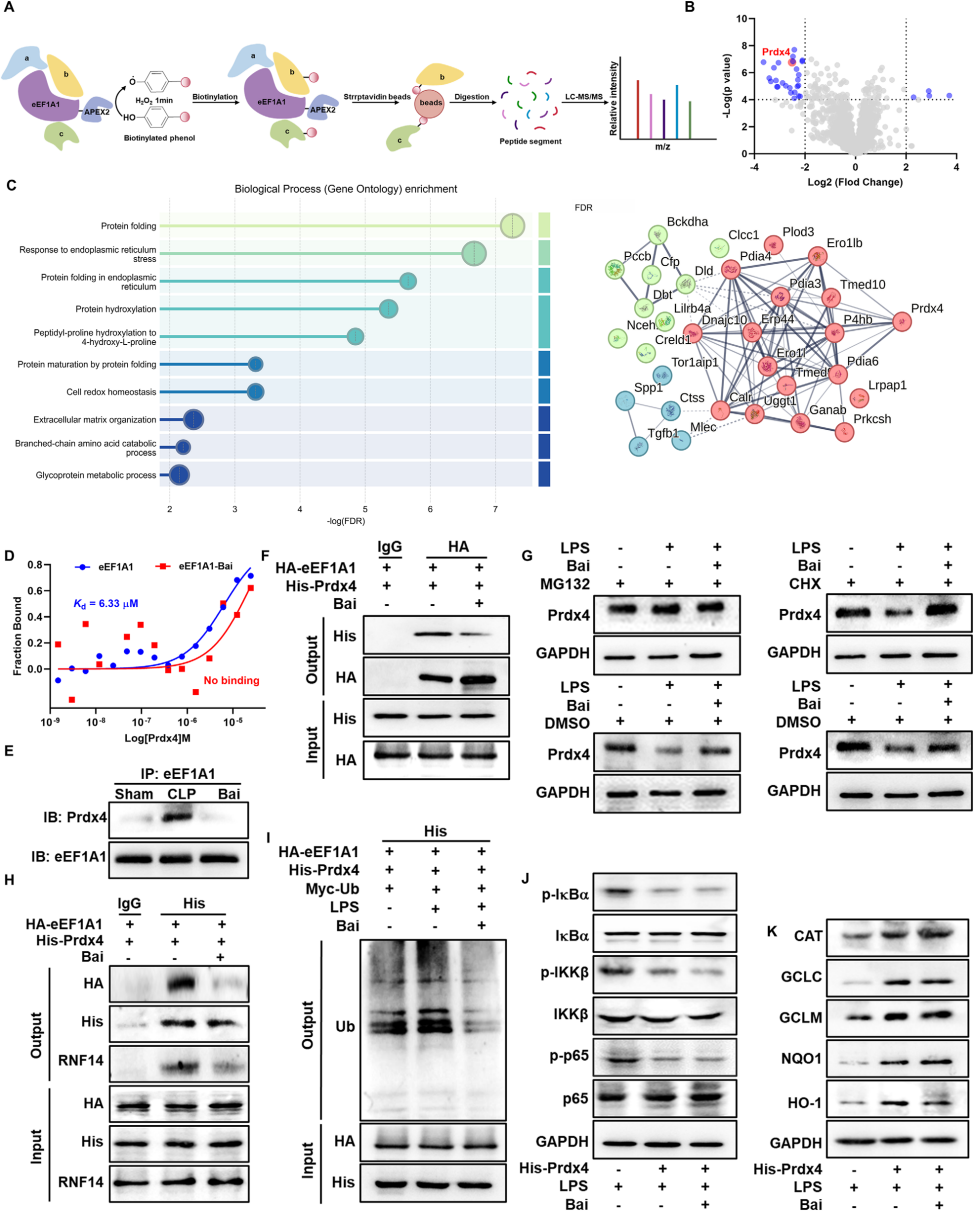
Bai blocked the interaction of eEF1A1 with Prdx4 to suppress the ubiquitylation and degradation of Prdx4. (A) Schematic of APEX2‐mediated proximity labeling assay; (B) Prdx4 was identified as the key interaction protein of eEF1A1; (C) GO enrichment and protein and protein interaction analysis; (D) MST plot (n = 3); (E,F) Bai blocked the interaction of eEF1A1 with Prdx4 in vitro and in vivo (*n* = 3); (G) Effects of Bai toward Prdx4 in the presence of CHX or MG132 in LPS‐stimulated cells (*n* = 3); (H) The binding of Bai with eEF1A1 blocked the interaction of Prdx4 with RNF14 (*n* = 3); (I) Ubiquitylation assay of Prdx4 (*n* = 3); (J,K) Prdx4 overexpression abolished the inhibitory effect of Bai toward inflammation and oxidative stress (*n* = 3); *p < 0.05, **p < 0.01, ***p < 0.001, ns = no significance.

Next, we performed the APEX2 proximity labeling experiment to elucidate the mechanism of eEF1A1 in inflammation (Figure 6A). The LC‐MS/MS results indicated the presence of 31 differential proteins in the APEX2 pull down experiment after Bai treatment (Figure 6B). GO and PPI net analyses (Figure 6C) illustrated that Prdx4 was the key interaction protein of eEF1A1 that was involved in inflammation and oxidative stress. Based on these findings, we further examined the molecular interplay between eEF1A1 and Prdx4 in modulating inflammatory response. MST and Co‐IP assays confirmed a direct physical interaction between the two proteins, and Bai could block their interaction in vitro and in vivo (Figure 6D–F). Furthermore, Bai repressed the LPS‐mediated Prdx4 decrease, while this effect was blocked after MG132 pretreatment rather than cycloheximide (CHX) pretreatment (Figure 6G), which indicated that its decrease involved proteasome‐dependent degradation. As shown in Figure 6H, our APEX2 proximity labeling result identified RNF14, which was an E3 ubiquitin ligase previously reported to interact with eEF1A1. eEF1A1 mediated the recruitment of RNF14 to Prdx4, while Bai disrupted this eEF1A1‐dependent Prdx4‐RNF14 interaction, thereby effectively suppressing RNF14‐mediated Prdx4 ubiquitylation and degradation (Figure 6H,I).

**FIGURE 7:**
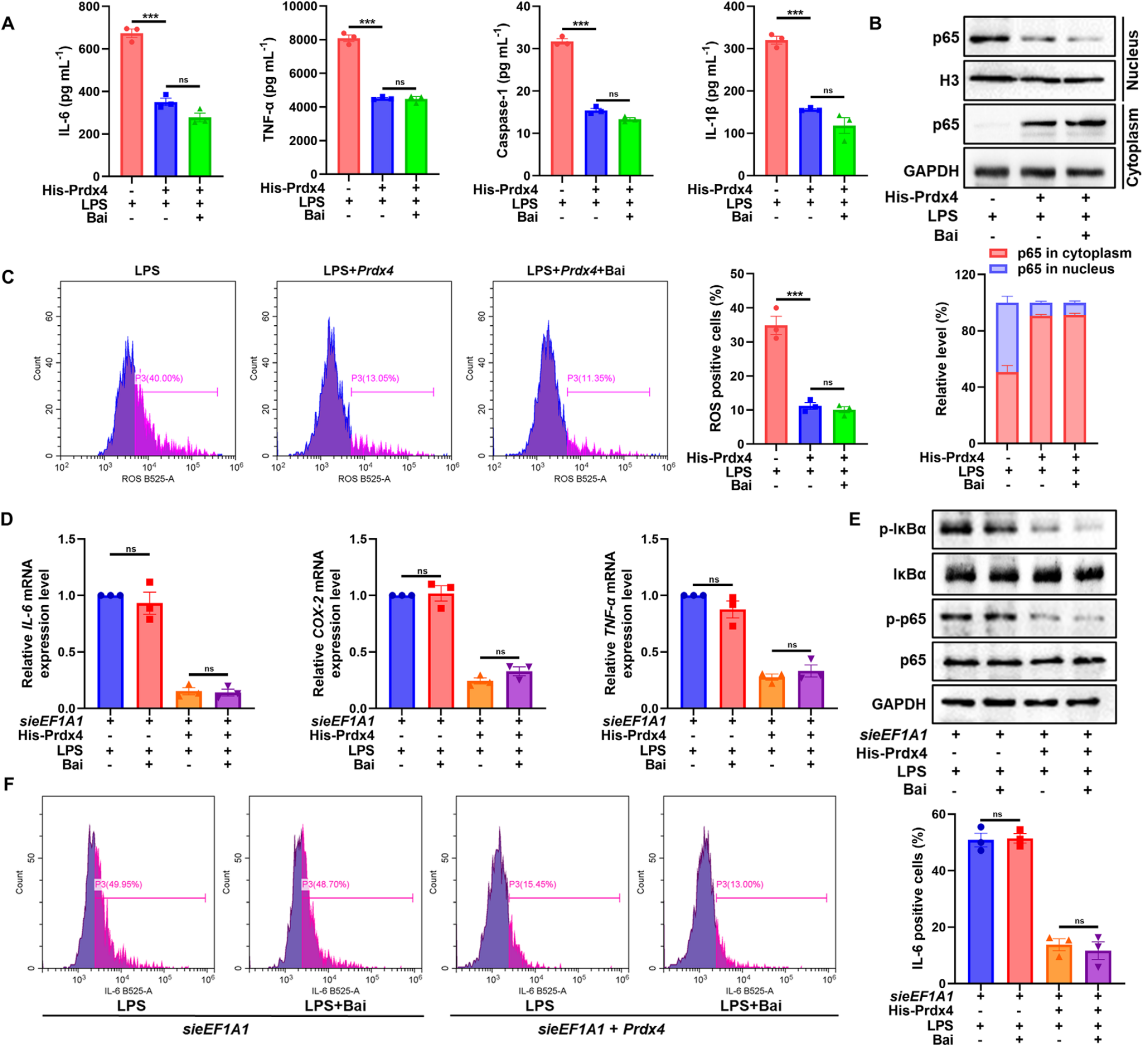
Anti‐inflammatory and antioxidant effects of Bai were dependent on eEF1A1‐Prdx4. (A) Prdx4 overexpression abolished effects of Bai on IL‐6, TNF‐α, IL‐1β, and caspase‐1 (– 3); (B) Overexpression of Prdx4 blocked Bai‐mediated suppression of p65 nuclear translocation (*n* = 3); (C) Flow cytometry results of ROS (*n* = 3); (D) Replenishment of Prdx4 after sieEF1A1 eliminated effects of Bai on mRNA levels of IL‐6, TNF‐α, and COX‐2 (*n* = 3); (E) Prdx4 rescue eliminated the inhibitory effect of Bai in NF‐κB pathway after sieEF1A1 (*n* = 3); (F) Flow cytometry results of IL‐6 in LPS‐mediated cells after Prdx4 rescue and sieEF1A1 (*n* = 3); *p < 0.05, **p < 0.01, ***p < 0.001, ns=no‐significance.

To assess the functional role of Prdx4 as the key downstream protein of eEF1A1 in Bai‐mediated anti‐inflammatory and antioxidant effects, Prdx4 plasma was transfected into LPS‐exposed RAW264.7 cells (Figure S7). Prdx4 overexpression suppressed the NF‐κB activation and increased expression levels of key antioxidant enzymes (e.g., CAT, GCLC, GCLM, NQO1, and HO‐1) (Figures 6J,K and 7C; Figure S8A,B). Meanwhile, its rescue abrogated the inhibitory effects of Bai on IL‐6, TNF‐α, IL‐1β, caspase‐1 secretion, and ROS production (Figure 7A–C). Anti‐inflammatory and antioxidant effects of Bai were critically dependent on the eEF1A1‐Prdx4 axis, as evidenced by the loss of its suppressive activity on IL‐6, TNF‐α, COX‐2, and p‐p65 following eEF1A1 knockdown and Prdx4 overexpression in vitro (Figure 7D–F). Collectively, Bai could block the eEF1A1‐mediated interaction of Prdx4 with RNF14 to suppress the RNF14‐mediated ubiquitylation and degradation of Prdx4.

### 3.7 eEF1A1 Knockdown Abolished the Anti‐ALI Effect of Bai In Vivo

**FIGURE 8:**
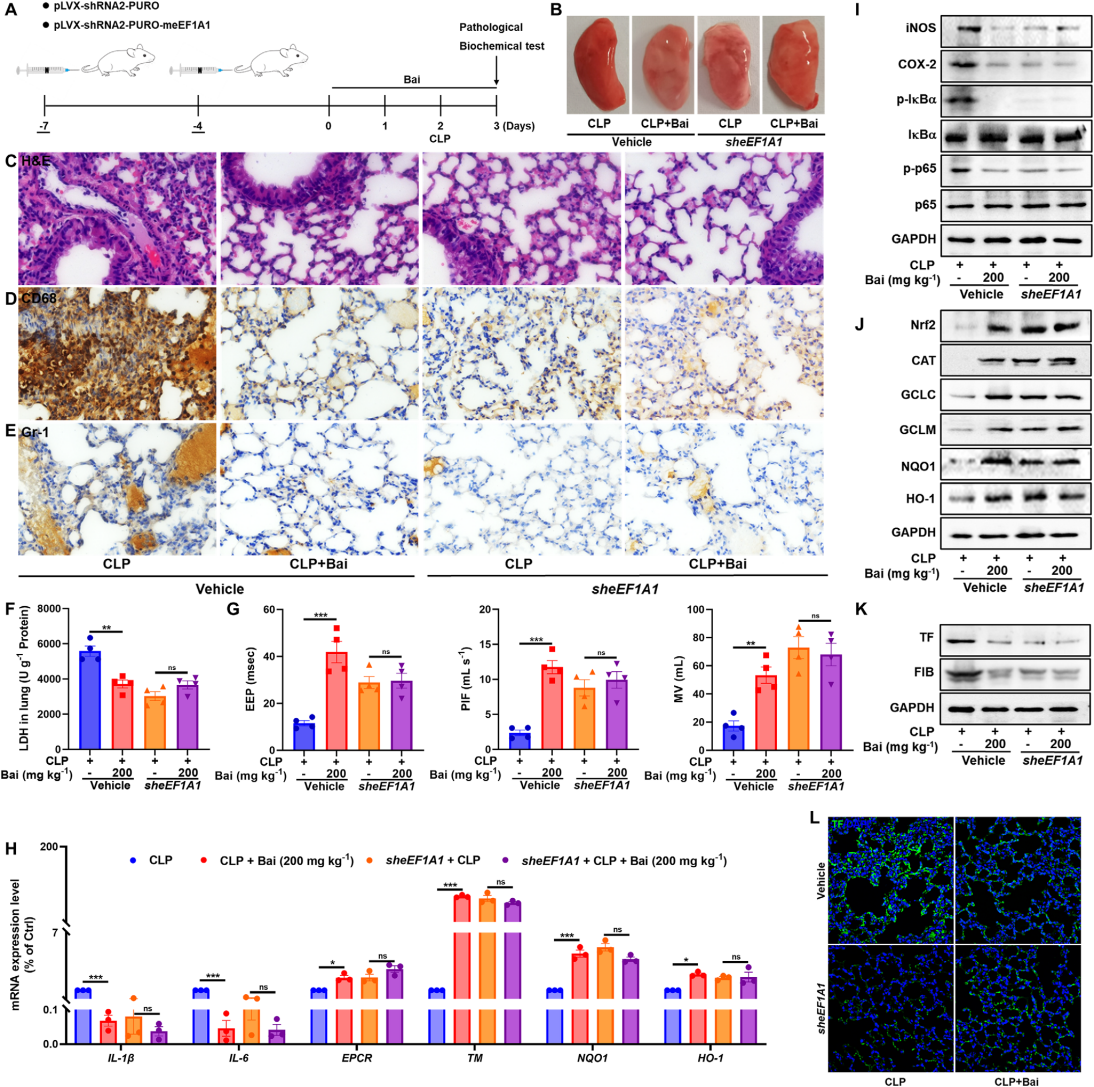
eEF1A1 knockdown abolished the protective effect of Bai on sepsis‐induced lung injury. (A) Schematic diagram of CLP‐mediated lung injury in eEF1A1 WT and knockdown mice; (B) Representative images of lung tissues (*n* = 4); (C‐E) Representative H&E, CD68, and Gr‐1 staining plots (*n* = 3); (F) LDH activity in eEF1A1 WT and knockdown mice (*n* = 4); (G) Pulmonary function measured by whole‐body plethysmography (*n* = 4); (H) Effects of Bai toward mRNA expressions of IL‐6, IL‐1β, NQO1, HO‐1, TM, and EPCR in CLP‐mediated eEF1A1 WT and knockdown mice (*n* = 3); (I,J) eEF1A1 knockdown abolished anti‐inflammatory and antioxidant effects of Bai (*n* = 3); (K) Effects of Bai toward TF and FIB in CLP‐mediated eEF1A1 WT and knockdown mice (*n* = 3); (L) Representative TF staining plots (*n* = 3); *p < 0.05, **p < 0.01, ***p < 0.001, ns = no‐significance.

To validate the in vivo relevance of the aforementioned mechanism, eEF1A1 was knocked down via lentiviral delivery in CLP‐induced ALI mice (Figures 8A; Figure S9). eEF1A1 knockdown alleviated pulmonary LDH release, lung morphology, and multiple pulmonary function parameters (e.g., EV, TI, and TV), indicating the key role of eEF1A1 in ALI, while Bai did not have further effects in eEF1A1‐deficient mice (Figure 8B,F–G; Figure S10C). Figure 8C–E showed that the sheEF1A1 group had thinner alveolar walls, decreased inflammatory cell infiltration, and inactivated macrophages and neutrophils. Meanwhile, its knockdown suppressed the coagulation factors (e.g., TF and FIB), inflammation (e.g., IL‐6, IL‐1β, and NF‐κB), and oxidative stress (e.g., Nrf2, CAT, GCLC, GCLM, NQO1, and HO‐1) in vivo (Figure 8H–L; Figure S10A,B). Notably, Bai did not further exert protective effects for these indices in CLP‐mediated eEF1A1 knockdown mice, illustrating that its protective effects depended on eEF1A1 in vivo.

## 4 Discussion

**FIGURE 9:**
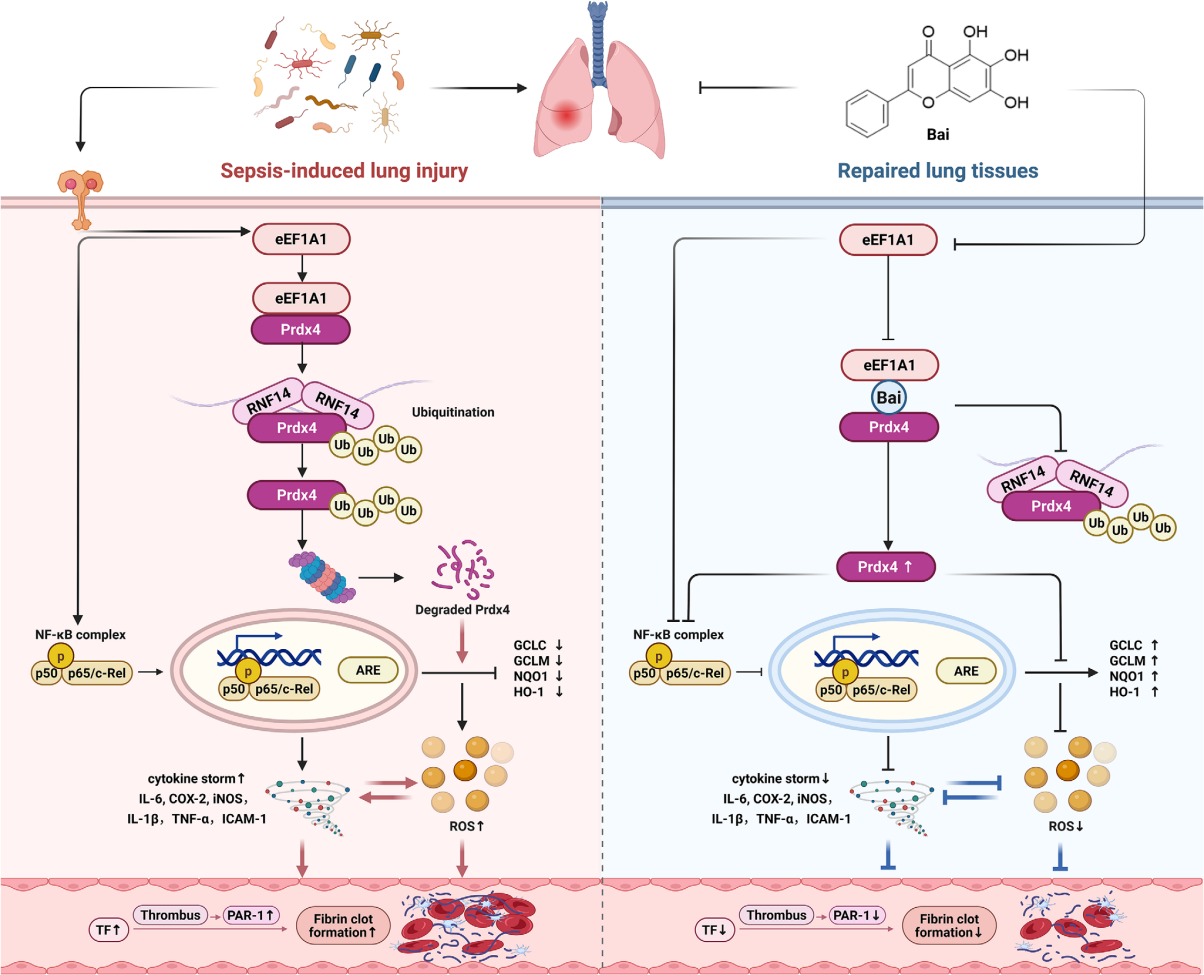
Mechanism of action of Bai in the treatment of sepsis‐mediated lung injury via targeting eEF1A1 to block eEF1A1/Prdx4 interaction.

Sepsis‐induced lung injury represents a major cause of death in patients with severe sepsis/septic shock [41]. Sepsis‐induced lung injury involves excessive inflammation, oxidative stress, and coagulation dysfunction, which collectively drive lung tissue deterioration [14, 42, 43]. In this pathogenic cascade, inflammatory signaling promotes coagulation and oxidative stress, while oxidative damage further amplifies endothelial dysfunction, forming a vicious cycle that culminates in organ failure [44, 45]. Our study demonstrated that Bai markedly ameliorated coagulation factor dysregulation in septic mice, thereby restoring pulmonary function and tissue architecture. Bai also suppressed aberrant activation of the NF‐κB pathway, reduced the release of pro‐inflammatory cytokines, and limited macrophage and neutrophil infiltration, thus contributing to the maintenance of pulmonary immune homeostasis. Mechanistically, Bai exerted its effects by binding to eEF1A1 and blocking the interaction of eEF1A1 and Prdx4 to inhibit RNF14‐mediated ubiquitylation and degradation of Prdx4. These findings highlighted the potential of developing modulators targeting the eEF1A1‐Prdx4 axis as therapeutic agents for sepsis and related disorders (Figure 9). Furthermore, our study indicated that eEF1A1 knockdown alleviated the course of sepsis‐induced lung injury, with no additional effects observed for Bai. These findings provided therapeutic avenues in the treatment of ALI by targeting eEF1A1, while also emphasizing the potential of Bai as an eEF1A1 modulator.

Natural products offered a distinct advantage in anti‐inflammatory drug discovery due to their inherent structural complexity and bioactivity [46, 47, 48, 49, 50, 51, 52, 53, 54]. Modern pharmacological research has demonstrated that *Scutellariae Radix* and its formulations, such as tablets and capsules, exhibited broad‐spectrum anti‐inflammatory, antioxidant, and antimicrobial activities, supporting their clinical application in respiratory and gastrointestinal disorders [31, 55]. Metabolic studies showed that baicalin, one of the main components of Scutellariae Radix, were prodrugs, which were converted into Bai by enzymatic hydrolysis in vivo [56, 57]. Pharmacological studies suggested Bai suppressed neuroinflammation via down‐regulation of NF‐κB and AMPK/Nrf2 pathways in ischaemic/reperfusion injury [58]. In LPS‐induced ALI, Bai reduced pro‐inflammatory cytokine levels by targeting both NF‐κB and MAPK pathways [36, 37]. As previously reported, we also found that Bai exerted protective effects against sepsis‐mediated lung injury, thereby exerting anti‐inflammatory, antioxidant, and anticoagulant effects.

Despite rapid progress in target screening technologies, the identification of specific drug targets remains difficult [16, 59, 60]. Methods including affinity chromatography, biotin‐avidin interaction, and stability shift‐based proteomics have successfully facilitated the discovery of direct targets for numerous natural products [61, 62, 63, 64]. In this study, employing affinity chromatography, we identified eEF1A1 as the direct cellular target of Bai. The translation factor eEF1A1, responsible for protein synthesis, is also implicated in viral replication and cancer progression [65]. Elevated eEF1A1 expression is closely associated with poor prognosis in colorectal and liver cancers, as well as chronic conditions like neuroinflammation [25, 65, 66]. Some evidence has illustrated that eEF1A1 silencing suppressed the NF‐κB pathway and pro‐inflammatory cytokine production [28, 29], and its binding ligand neferine could suppress M1 macrophage polarization in atherosclerosis [47], indicating its potential in inflammatory response. As expected, eEF1A1 knockdown attenuated inflammatory response in the LPS‐mediated cellular model and the sepsis‐induced ALI mouse model, while this effect was reversed after eEF1A1 rescue. eEF1A1 knockdown and rescue abolished Bai's benefits, providing direct evidence that its efficacy relied on this target.

This study employed APEX2‐mediated proximity labeling, a cutting‐edge technique that enables the spatiotemporal capture of protein‐protein interactions within living cells [21, 67]. This powerful approach allowed us to map the native interactome of eEF1A1 in macrophages, directly leading to the identification of Prdx4 as its critical binding partner and downstream effector. Interrogation of the eEF1A1 interactome via APEX2 proximity labeling yielded the definitive identification of Prdx4. Beyond its antioxidant role, Prdx4 exerted anti‐inflammatory effects by directly interacting with caspase‐1 [68, 69]. This interaction inhibited caspase‐1 activity, leading to reduced IL‐1β maturation and secretion, which underpinned the heightened sensitivity of Prdx4‐deficient mice to septic shock [69]. In this study, we first reported that Bai could block the interaction of eEF1A1 and Prdx4 to suppress RNF14‐mediated ubiquitylation and degradation of Prdx4. Meanwhile, Bai did not show extra effects in Prdx4 overexpression cells, which suggested that Prdx4 was the key downstream of eEF1A1 for treating sepsis‐mediated lung injury.

## 5 Conclusion

Collectively, pioneering our investigation, we identified eEF1A1 as an unprecedented approach to target the polymorphic pathology of sepsis through interacting with Prdx4 and regulating RNF14‐mediated ubiquitylation and degradation of Prdx4. The small‐molecule inhibitor Bai exerted its effect by binding to Q108 and D110 of eEF1A1, thereby stabilizing Prdx4 by blocking its ubiquitylation and degradation. These findings indicated that eEF1A1 inhibition, alongside its ligand Bai, targeted septic lung injury, highlighting its therapeutic value.

## Author Contributions

**Jing Chang**: investigation, visualization, software, writing – original draft, writing – revise and editing. **Hui‐Lin Zhang**: investigation, visualization, writing – original draft, writing – revise and editing. **Qi‐Meng Zhu**: investigation, visualization. **Si‐Wen Hui**: investigation. **Xin‐Rong Xu**: investigation. **Mahmood Brobbey Oppong**: investigation. **Yan‐Li Feng**: software. **Juan Zhang**: conceptualization, writing – original draft, writing – revise and editing, supervision. **Cheng‐Peng Sun**: conceptualization, writing – original draft, writing – revise and editing, supervision. **Feng Qiu**: conceptualization, writing – original draft, writing – revise and editing, supervision.

## Conflicts of Interest

The authors declare no conflicts of interest.

## Ethics Statement

All experimental procedures were approved by the Animal Care and Use Committee of Tianjin University of Traditional Chinese Medicine (No. TCM‐LAEC2023208s1550).

## Supporting information




**Supporting File**: exp270206‐sup‐0001‐SuppMat.docx.

## Data Availability

Data supporting the results in this work are available from the corresponding authors upon reasonable request.
