# Supplementary material for: Targeting eEF1A1 With Baicalein to Block eEF1A1/Prdx4 Interaction for Treating Sepsis‐Mediated Lung Injury
Source: Exploration (Beijing). 2026 Aug 24;6(5):70206. doi: 10.1002/exp2.70206 (PMC13503625; doi:10.1002/exp2.70206)
Supplement: Supplementary file 1 [file EXP2-6-70206-s001.docx]

Supplementary Information Text

**Figure S1. Bai displayed anti-inflammatory and oxidative stress potential *in vitro*.** (A) Effects of Bai toward cell viability (n = 9); (B) Flow cytometry quantization of IL-6, TNF-α and ROS (n = 3); (C) Protein quantification of COX-2, iNOS, p-p65, p-IKKβ, and p-IκBα in RAW 264.7 cells induced by LPS (n = 3); (D) Protein quantification of Nrf2, CAT, GCLC, GCLM, HO-1, and NQO1 in RAW 264.7 cells induced by LPS (n = 3 **p* < 0.05, ***p* < 0.01, ****p* < 0.001, ns=no-significance).

**Figure S2. Effects of LPS toward cell viability.** Effects of LPS toward cell viability (n = 6 **p* < 0.05, ***p* < 0.01, ****p* < 0.001, ns=no-significance).

**Figure S3. Bai displayed anti-inflammatory and oxidative stress potential in LPS-induced MH-S cell.** (A) Bai dose-dependently suppressed the LPS-induced mRNA expression of *NQO1*, *IL-1β*, *COX-2* and *CXCL10* (n = 3); (B) Effects of Bai toward Nrf2, iNOS, GCLM, p-p65 and p65 in LPS-induced MH-S cell (n = 3); (C) Protein quantification of Nrf2, iNOS, GCLM, and p-p65 (n = 3; **p* < 0.05, ***p* < 0.01, ****p* < 0.001, ns=no-significance).

**Figure S4. Bai regulated coagulation-regulating, anti-inflammatory, and oxidative stress effects in sepsis-induced lung injury.** (A) Bai suppressed mRNA levels of coagulation factor *TM* in CLP-mediated lung injury (n = 3); (B) Effects of Bai on the expression of TF and FIB in sepsis-induced lung injury by CLP (n = 3); (C) Bai suppressed mRNA levels of inflammation factor *iNOS* in CLP-mediated lung injury (n = 3); (D) Protein quantification of iNOS, COX-2, p-p65, p-IKKβ, and p-IκBα in sepsis mice (n = 3); (E) The expression levels of Nrf2, GCLM, GCLC, HO-1, and NQO1 in CLP-induced lung injury (n = 3); (F) Representative iNOS staining plots (n = 3; **p* < 0.05, ***p* < 0.01, ****p* < 0.001, ns=non-significant).

**Figure S5.  The expression levels of eEF1A1 in RAW 264.7.** Effects of Bai toward eEF1A1 in LPS-induced RAW264.7 (n = 3; **p* < 0.05, ***p* < 0.01, ****p* < 0.001, ns=non-significant).

**Figure S6.  The inhibitor of eEF1A1, plitidepsin, abolished the effects of Bai *in vitro*.** (A) Effects of Bai toward cell viability (n = 6); (B) The expression levels of p-p65, GCLM, and iNOS in RAW 264.7 (n = 3; **p* < 0.05, ***p* < 0.01, ****p* < 0.001, ns=no-significance).

**Figure S7.  Western blot verified overexpression of *Prdx4*.**

**Figure S8.  *Prdx4* overexpression abolished anti-inflammatory and oxidative stress effects of Bai *in vitro*.** (A) Protein quantification of p-p65, p-IKKβ, and p-IκBα in LPS-induced RAW 264.7 cell (n = 3); (B) The expression levels of NQO1, GCLC, CAT, HO-1, and GCLM in RAW 264.7 cell induced by LPS (n = 3; **p* < 0.05, ***p* < 0.01, ****p* < 0.001, ns=no-significance).

**Figure S9.** **Confirmation of *eEF1A1* knockdown mice**

**Figure S10. *eEF1A1* knockdown abolished the protective effect of Bai on sepsis-induced lung injury.** (A) Protein quantification of Nrf2, HO-1, CAT, GCLM, NQO1, and GCLC in CLP-mediated *eEF1A1* WT and knockdown mice (n = 3); (B) The expression levels of iNOS, COX-2, p-p65, and p-IκBα (n = 3); (C) Pulmonary function indices measured by whole-body plethysmography in CLP-mediated *eEF1A1* WT and knockdown mice (n = 4; **p* < 0.05, ***p* < 0.01, ****p* < 0.001, ns=no-significance).
